# Introducing a Board of Associate Reviewers

**DOI:** 10.1523/ENEURO.0184-26.2026

**Published:** 2026-07-08

Peer review has long been a cornerstone of scientific advancement, upholding rigorous standards and helping to maintain public trust in the scientific process. Through independent expert evaluation, peer review helps ensure that research methods are sound, analyses are appropriate, and conclusions are supported by the evidence. Research that has been peer-reviewed comes with a promise—that it is reliable, accurate, and to be trusted.

Yet, unpublished manuscripts are reviewed by active researchers recruited on an ad hoc basis, which makes securing reviewers harder than ever. While reviewers are chosen for their expertise, there is no guarantee whether they will accept the reviewing assignment. Alongside running intensive research programs, many are expected to teach in the classroom, mentor the new generation of researchers, and engage in service to their institution, the agencies that fund their research, the journals that publish their work, and the list goes on. It is therefore not surprising that finding time to review manuscripts can be challenging. This is compounded by ever-growing submissions, further increasing demand for researchers to evaluate their peers’ work.

As a result, editors are often required to send a large number of requests to review, many of which are declined or go unanswered. As invitations extend further down the reviewer list, the match between reviewer expertise and manuscript content may weaken. This can lead to reviews that are tangential or insufficiently informed, which can be frustrating for authors and can ultimately undermine the quality and efficiency of the review process. Thus, the process of securing reviewers ad hoc brings unpredictability and inconsistency to the review process that is not always appreciated by any of the parties involved.

To ensure that manuscripts submitted to *eNeuro* receive fair, appropriate, and timely assessment, we are introducing a standing board of Associate Reviewers. This board comprises over 40 active scientists recognized by our Reviewing Editors, Advisory Board, and Editors-in-Chief for their consistency in providing in-depth, informed, and timely manuscript assessments for *eNeuro* and elsewhere. The expertise of our board is broad and spans all areas of neuroscience, to reflect the diversity of submissions that *eNeuro* receives.

Having a board of dedicated, accountable reviewers serves a clear purpose. It allows *eNeuro* to better detect flawed methods, flag inadequate reporting, and identify questionable claims. It also ensures consistency during the review process across submissions. The regular interaction between the reviewers and editors will ensure that everyone is trained and accountable, that our board is calibrated, and expectations regarding manuscript requests are similarly applied across all manuscripts. More time during the review process can be devoted to the collaboration between the reviewers and editors on providing clear, justified, and actionable recommendations for publication to the authors, furthering the journal's aim to reduce the noise in peer review as well as unnecessary rounds of revision. Thus, this process will ensure reliability, provide quality control, and build trust.

Our standing board of reviewers who will work alongside our ongoing use of ad hoc reviewers will provide an optimal balance between preserving broad community input and access to appropriate technical expertise, while simultaneously reducing administrative burden and improving efficiency. Thus, when submitting to *eNeuro*, authors can be confident that their manuscript will be appropriately evaluated by some of the best experts in the field and thus receive high-quality, fair, and rapid reviews that make only critical requests for additional work. In doing so, we strive for *eNeuro* to provide authors with a consistently positive review experience that is upheld across all submissions.

Reviewers do essential, irreplaceable work and deserve meaningful professional recognition. *eNeuro* will officially recognize and give visibility to these reviewers by incorporating them into the journal's editorial structure, and their names, affiliations, and expertise will appear on the journal’s editorial board page. To further recognize their contributions and service to science, the Editors-in-Chief are committed to providing support for the professional advancement of Associate Reviewers within the journal structure as well as in the form of recommendation letters for promotion or award applications. In addition, as a Society for Neuroscience journal, *eNeuro* will offer Associate Reviewers attendance perks for the Society's annual meeting.

With the introduction of the board of Associate Reviewers, we aim to strengthen peer review at *eNeuro*, enhance the author experience, and recognize the vital contributions of reviewers.

Please welcome Associate Reviewers Dominic Acri, Hossein Aleyasin, Brian Allman, Benedicte Amilhon, Sufyan Ashhad, Estelle Barbier, Pedro Bekinschtein, Nicole Bentley, Christian Bravo, Yarimar Carrasquillo, Bharath Chelluboina, Rupesh Chillale, Jonathan Chow, Anindita Das, Giannina Descalzi, Ginevra D'Ottavio, Ulrich Egert, Matthew Gardner, Nicolas Gervasi, Sabina Gherman, Jackie Giovanniello, Evan Hart, Rutsuko Ito, Abhilasha Joshi, Ron Keiflin, Lori Knackstedt, Belinda Lay, Xiaoyu Ma, Fulin Ma, Vincenzo Mastrolia, Ciaran Murphy-Royal, Yulia Oganian, Martin Orf, James Otis, Shauna Parkes, Polyxeni Philippidou, Eugene Poh, Raghav Rajan, Raj Rajebhosale, Leslie Ramsey, Simone Russo, Giriraj Sahu, Hillary Schiff, Roberto Vincis, Jai Yu, and Natalie Zlebnik.

